# Delegate contract signing mechanism based on smart contract

**DOI:** 10.1371/journal.pone.0273424

**Published:** 2022-08-19

**Authors:** Wei Xiong, Yangcheng Hu

**Affiliations:** School of Business Administration, Nanchang Institute of Technology, Nanchang, 330099, China; Dai Hoc Duy Tan, VIET NAM

## Abstract

In this paper, a delegate contract signing solution is proposed to eliminate the potential risk of contract fraud caused by information and interest asymmetry. By utilizing the functional properties of the Ethereum blockchain and smart contracts, a delegate contract signing mechanism is established. By running the mechanism, the delegate contract signing information is received and processed, and the information is broadcast to the blockchain network nodes. By designing the algorithms of "requesting contract signing", "successful contract signing" and "contract fraud dispute resolution", the delegate contract signing is realized. By proposing algorithms and their calling processes, the smart contracts are completed. Finally, the smart contracts based on the solution are tested and verified. The source code of the smart contracts has been published on GitHub.

## Introduction

Delegate authorization is usually used to judge the eligibility of the sales manager to sign the financial investment contract with the client. Such mechanism is modeled as the delegate-based contract signing (DBCS) framework, which depicts the financial investment contract created and signed relation between the sales manager and the client. In the DBCS, the sales manager is related to the delegate authorization, and the delegate authorization is related to the client. This framework enables the establishment of the delegate contract signing mechanism between the sales manager and the client. For example, the financial investment contract is designed as the financial investment smart contract [1], and the delegate authorization certificate is designed as the delegate authorization smart contract, and they are published in a public medium. The client can access the smart contracts through the public medium, and then verify that the sales manager is qualified to sign the financial investment contract with the client and check the content of financial investment contract.

To achieve the DBCS mechanism in the blockchain [2], the client can verify the financial investment contract content and the delegate authorization of the sales manager. The sales manager cannot disguise his/her delegate authorization, that is, the sales manager cannot use the delegate authorization that he/she does not own. Currently, there is a problem of contract fraud [3]. For example, financial investment advisors want clients to bind their interests, and financial investment contract issuing companies also want clients to bind their interests. Due to the asymmetry of information and interests between the client and the advisor, this can easily lead to contract fraud. Furthermore, advisors and clients can sign electronic contracts. However, due to the existence of a centralized third-party platform, this can easily lead to fraudulent behaviors such as data and signature tampering. On the other hand, in the process of financial investment, there is a problem of paying taxes [4]. To evade taxes, the financial investment advisor signs a "yin-yang contract" with the client. In other words, the small-value contract is used as the basis for tax payment, and the large-value contract is kept confidential.

For this reason, the following questions are raised in this paper: How to effectively implement the DBCS mechanism in a transparent manner? How to safely verify the delegate authorization of the sales manager of the contract-issuing organization? How to let the sales manager flexibly complete the control of his/her delegate authorization, and provide the contract-issuing organization with the flexibility to effectively manage its (sales manager)-delegate authorization? What other benefits can the DBCS mechanism provide?

A delegate-based contract signing using smart contracts (DBCS-SC) is presented. It achieves an DBCS mechanism using blockchain and smart contracts. Blockchain is first introduced by Bitcoin [2], which originally aim to achieve a secure and decentralized payment system and complete digital funds. The blockchain is a peer-to-peer network driven by users, without central management. With the implementation of Bitcoin and its underlying technology blockchain, many cryptocurrencies based on blockchain have emerged, including Ethereum [5], Litecoin [6], and Dogecoin [7]. In this paper, Ethereum is used to implement the prototypes of the smart contracts of the DBCS-SC. It is an open blockchain platform that allows the deployment of smart contracts. Smart contracts are self-executing scripts triggered by transactions and messages. The smart contracts of the DBCS-SC are written in Solidity [8], tested and compiled in Remix IDE [9], and verified in Ethereum Wallet [10].

This paper aims to implement an DBCS mechanism. The mechanism is safe (sales managers cannot disguise their delegate authorization and only authorized entities can perform functions), (sales manager)-oriented (sales managers can disclose their delegate authorization to any client and only authorized delegates can sign financial investment contracts with clients), verifiable (anyone can verify whether the sales manager has the delegate authorization managed and issued by the contract-issuing organization), and manageable (delegate authorization can be revoked and modified). The key idea is to issue financial-investment smart contract related to clients and delegate-authorization smart contract related to sales managers. The smart contracts are compiled and deployed on the Ethereum blockchain. The client can use the challenge-response agreement to verify that the sales manager is qualified to sign the financial investment contract with him/her.

The main contributions of this paper are summarized as follows:

1. The DBCS mechanism based on smart contracts is constructed to prevent contract fraud caused by information asymmetry and interest asymmetry.
2. The DBCS-SC framework is designed based on the challenge-response mechanism, and its performance, strengths and weaknesses are analyzed based on the features of blockchain and smart contracts.
3. It offers a new method for solving financial investment contract fraud caused by information asymmetry and interest asymmetry, and also supplies a new solution for the prior-prevention and post-regulation of financial investment contract fraud.
4. The prototypes of the DBCS-SC smart contract are implemented and deployed on the Ethereum blockchain. The source code of the prototypes is released on GitHub (https://github.com/106968687/DBCS-SC).

The rest of the paper is arranged as follows: Section 2 sorts out and analyzes the related work. Section 3 introduces the DBCS model and the problem statement. Section 4 designs and implements the DBCS-SC. Section 5 presents the implementation of the smart contracts. Section 6 shows the testing and verification of the smart contracts. Section 7 analyzes the performance, strengths and weaknesses of the DBCS-SC. Finally, the full text is concluded.

## Related work

The representative researches on blockchain-based contract signing and contract signing are summarized as follows.

### Blockchain-based contract signing

Mahajan et al. proposed a fusion method for global optimization tasks, which can be combined with blockchain to solve the problem of consistent balance and optimization for high-quality contract signing [11–13]. Ferrer-Gomila et al. proposed a multi-party contract signing protocol based on blockchain to ensure that when signing multi-party contracts, either all signatories obtain signing evidence or no one obtains conflict evidence of honest signatories, so as to solve the problem of fair exchange [14]. Zhang et al. proposed a safe, convenient, and blockchain-based way to sign private equity contracts to solve the problem of sharing private information among stakeholders and prevent forged signatures, forged seals and dual contracts [15]. Zhang et al. proposed a two-party fair contract signing scheme based on Ethereum smart contract to solve the problem that a centralized trusted third party may leak the content of the contract, collude with others, and even cause service interruption [16]. Ferrer-Gomila et al. proposed to use blockchain to sign contracts, avoid using centralized trusted third parties in a simple way, and employ any blockchain solution to enable centralized applications to find solutions to their problems [17]. Ferrer-Gomila et al. proposed a blockchain-based contract signing agreement to solve the problem of fairness, timeliness and non-repudiation of electronically signed contracts in e-commerce transactions [18]. Huang et al. proposed a fair tripartite contract signing agreement based on blockchain primitives to solve the problem of two or more mutually distrustful entities signing predefined digital contracts in a fair and effective manner [19]. Mut-Puigserver et al. proposed a contract signing agreement based on blockchain technology to solve the problem that contract signing usually relies heavily on the use of trusted third parties [20]. Wang et al. proposed a fair contract signing agreement based on blockchain privacy protection to ensure fair and just transactions between untrustworthy parties [21]. Wu et al. proposed a fair contract signing agreement based on a blind verification encryption signature scheme to solve the problem that the fairness and privacy of contract signing depend on the reliability of a third party [22]. Tian et al. proposed a blockchain-based contract signing application to solve the problem of fairness and privacy in the signed contract [23]. Huang et al. proposed a fair three-party contract signing agreement based on blockchain primitives to solve the problem of signing a predefined digital contract in a fair and effective manner without a trusted third party [24].

Scholars have made many contributions to the research of blockchain-based contract signing. However, there is another aspect that has not been researched, that is, delegate contract signing mechanism based on smart contract. The mechanism designs financial investment contracts as smart contracts, and delegate authorization contracts as smart contracts, and deploys them on the blockchain. It will ensure the integrity, non-repudiation and tamper resistance of financial investment contracts and delegate authorization contracts, as well as the transparency and traceability of contract signing information. This provides **a new method** to solve the contract fraud problem caused by information asymmetry and interest asymmetry.

### Contract signing

Mahajan et al. proposed a new method for contract selection based on cisoidal analysis to eliminate certain elements in the entire contract space to enhance contract selection and thus obtain a better contract for signing [25,26]. Xu et al. proposed an efficient contract signing agreement in the Internet of Things to solve the problem that the existing contract signing agreement cannot meet the requirements of future Internet of Things applications for efficiency and convenience [27]. Xu et al. proposed an abuse free contract signing protocol with low storage trusted third party to solve the problem of eliminating huge energy consumption from inappropriate protocol design [28]. Cao et al. proposed an efficient multi-party contract signing protocol to solve the security problem of signing contracts between two parties who do not trust each other [29]. Cai et al. proposed a new fair and optimistic contract signing protocol based on quantum cryptography to solve the problem of eliminating the signature exchange between two clients and reducing the complexity of communication [30]. Yadav et al. proposed a quantum scheme to solve the problem of signing a contract between two clients using entangled state and trusted third-party services [31]. Xiao et al. proposed a new concurrent signature and applied it to contract signing agreements to solve the problem of ensuring the fairness of contract signing [32]. Maimuţ et al. proposed a universal zero-knowledge co-signing agreement to solve the problem that there is no cornerstone for legal and fair contract signing [33]. Wang proposed an identity-based contract signing agreement based on bilinear pairings to solve two key issues of the practicality and efficiency of the contract signing agreement [34]. Maimuţ et al. proposed a trapdoor-based contract signing agreement to solve the problem that a secret embedded trapdoor mechanism with universal protection can be injected into a concurrent signature scheme and a legal and fair agreement without a cornerstone [35]. Zhai et al. proposed a new low-storage trusted third party and a non-abuse contract signing agreement to solve the problem of ensuring that the contract signing agreement is free from abuse and efficiency [36]. Ferradi et al. proposed a new contract signing paradigm that does not need a cornerstone to achieve legal fairness, so as to solve the problem of achieving fairness and ensuring output delivery in contract signing [37].

Scholars have made a lot of contributions to the research on contract signing. However, with the emergence of blockchain and smart contract technology, **a new solution** can be provided for the prior-prevention and post-regulation of contract fraud, that is, a (smart contract)-based blockchain solution for delegate contract signing. This solution will help protect the rights of participants and establish trust in any future events.

## Model for DBCS and problem statement

### Model for DBCS

In the DBCS model, the contract signing structure is defined by four sets (set *S* of sales managers, set *D* of delegates, set *C* of clients, and set *O* of contract-issuing organizations) and two relations ((sales manager)-delegate authorization *SA*⊂*S*×*A* and delegate-client contract signing *CS*⊂*C*×*S*). A sales manager *s* is eligible to sign contract with a client *c* if and only if there is a delegate *d* such that (*s*,*d*)∈*SA* and (*d*,*c*)∈*CS*. A sales manager with delegate authorization can sign financial investment contracts with clients in a safe and transparent way. The client entity can check the delegate authorization issued by the contract-issuing entity to determine whether it should sign an investment contract with an unknown sales manager. Moreover, the set *D* of delegates is broken up into several subsets, and each subset of *D* is associated with an element in *O*, that is to say, $D=D_{o_{1}}\cup \cdots \cup D_{o_{n}}$, $where\;o_{1},\cdots ,o_{n}\in O\;and\;D_{o_{i}}\cap D_{o_{j}}=\emptyset if\;i\neq j$. To clarify the relation between the organization and the delegate, the delegate *d* in $D_{o_{1}}$ is written as *o*_1_.*d*. Equally, the (sales manager)-delegate authorization *SA* is broken up into disjoint subsets, that is to say, $SA=SA_{o_{1}}\cup \cdots \cup SA_{o_{n}},where\;SA_{o_{i}}\subset S\times D_{o_{i}}$. Evidently, $o_{1}.d\in D_{o_{i}}$ means that the delegate *o*_1_.*d* is administered by the contract-issuing organization *o*_*i*_ and the assignment of sales managers to *o*_1_.*d* is regulated by the contract-issuing organization *o*_*i*_.

In the DBCS, the sales manager *s* requests the contract signing with the client *c* by declaring the delegate $o_{1}.d\in D_{o_{i}}$ which has been issued by the contract-issuing organization *o*_1_. The client signs the financial investment contract with the sales manager if and only if $(s,o_{i}.d)\in SA_{o_{i}}\;and\;(o_{i}.d,c)\in CS$. Remark that the client can be prone to verify (*o*_1_.*d*,*c*)∈*CS* because it defined *CS*. On the other hand, it cannot be prone to verify $(s,o_{i}.d)\in SA_{o_{i}}$, due to the contract-issuing organization defined $SA_{o_{i}}$. Sometimes this verification process is called identity authentication.

### Problem statement

In the DBCS, it is important to verify the authenticity of the (sales manager)-delegate authorization and the content of the financial investment contract. For example, a client should be able to verify whether a sales manager is the rightful owner of the declared delegate and whether the delegate is issued by the corresponding contract-issuing organization. The DBCS is going to be insecure and result in contract fraud without this verification process. In the process of signing a paper contract, due to the asymmetry of information and interests between the sales manager and the client, the sales manager may commit contract fraud to the client. In the process of signing an electronic contract, since the content and signature of the contract is in electronic format, it is easy to tamper with the content and signature. When signing the "yin-yang contract", there are two contracts to be signed, one is a large amount of money contract and the other is a small amount of money contract. Only the small amount of money contract is public to achieve the purpose of tax evasion.

This paper mainly implements the DBCS mechanism in a safe and transparent way. By designing the financial investment contract as a smart contract, the transparency and immutability of the contract content and signature is realized, so as to avoid the clients being cheated. By designing the delegate authorization certificate as a delegate authorization smart contract, the security and transparency of the delegate authorization can be realized. This helps the client verifies the authenticity of the delegate authorization of the unknown sales manager. Then, after the client verifies the delegate authorization of the unknown sales manager, the sales manager and the client can sign the financial investment smart contract. To fulfill these goals, the following attributes should be possessed by the proposed system:

1. Issuance: Contract-issuing organizations can issue financial investment contracts and delegate authorization contracts, such as contract content and authorization delegate.
2. Management: Contract-issuing organizations can manage and modify delegate authorization information as required in a transparent way.
3. Revocation: Contract-issuing organizations can revoke the delegate authorization of the sales manager as required in a transparent way.
4. Verification: Before the financial investment contract is signed, any entity can verify the delegate authorization of the unknown sales manager by the challenge-response agreement.
5. Transparency: All operations performed in the smart contracts are recorded, and these operations can be audited by any entity.
6. Restriction: Specific actions can only be performed by specific entities, and entities without authority cannot pretend to be authorized to perform specific actions.

## Delegate contract signing mechanism based on smart contract (DBCS-SC)

### Overview

**Fig 1:**
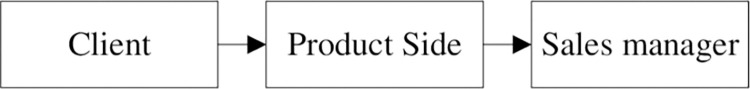
The value flow direction.

**Fig 2:**
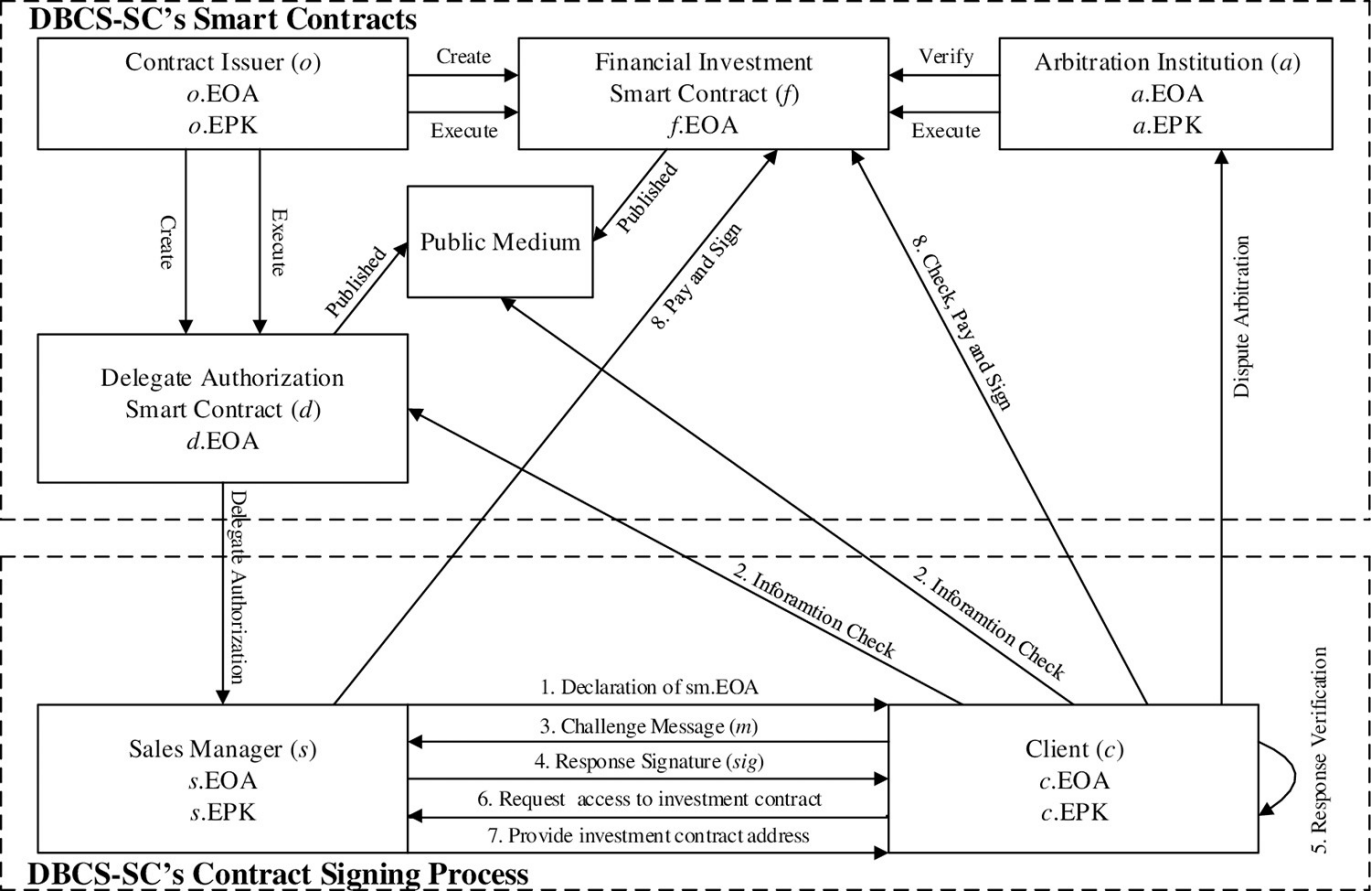
The framework of DBCS-SC.

This paper researches how to effectively implement the DBCS mechanism in a transparent and non-tampered way, and verify the delegate authorization of the contract-issuing organization (i.e. Product Side) to the sales manager in a secure manner. To achieve these goals, the DBCS-SC is presented. It is an DBCS mechanism based on smart contracts. This mechanism is suitable for signing financial investment contracts between the delegate and the client, which can effectively prevent contract fraud. Fig 1 shows the value flow direction. Fig 2 shows the complete structure of the proposed DBCS-SC system. The DBCS-SC is composed of two main parts, namely the smart contracts and the contract signing process. The participating entities in the DBCS-SC are summarized as follows:

1. Contract Issuer (*o*): A contract issuer is a professional contract-issuing organization that can create and deploy financial investment smart contracts and delegate authorization smart contracts.
2. Sales Manager (*s*): A sales manager is an entity that is authorized by the contract issuer to sign financial investment contracts.
3. Client (*c*): An entity that has a demand for financial investment and is willing to sign a financial investment contract through the delegate contract signing mechanism.
4. Arbitration Institution (*a*): An arbitration institution is an entity trusted by the contract issuer, sales manager, and client. The arbitration institution can confirm the correctness of the smart contract to ensure that the smart contract meets the interests of all participants. Moreover, it can resolve disputes among participating entities. It is assumed that the arbitration institution will comply with the principle of fairness and justice under all circumstances.

The financial investment smart contract (*f*) is used to create the content of the financial investment contract. The delegate authorization smart contract (*d*) is used to create the detailed information of the organization’s delegate authorization to the sales manager. They will be published on the blockchain. The smart contracts offer different functions to effectively, transparently and securely create contract content, (sales manager)-delegate authorization and contract signing. The use of blockchain can ensure the transparency of contract content, delegate authorization and all operations, while maintaining the anonymity of contract signing, and acting as a synchronization point for the client to check the declared delegate. A smart contract is a safe and efficient programmable asset that can be run accurately in a programmed manner. All functions performed using smart contract are automatically published on the blockchain. The features of the delegate authorization smart contract are as follows: (1) Allow the contract-issuing organization to authorize the delegate qualification to the sales manager; (2) Allow the contract-issuing organization to manage and modify the delegate authorization information as needed in a transparent way; (3) Allow the contract-issuing organization to revoke the delegate qualification authorized to the sales managers as needed. The characteristics of the financial investment smart contract are as follows: (1) Allow the contract-issuing organization to issue the financial investment contract content to the whole blockchain; (2) Allow the contract-issuing organization to add the signature of the contract signer to the financial investment contract; (3) Allow any entity to access and view the financial investment contract content.

The contract signing process is used to identify the authenticity of the contract content and verify the delegate authorization of the sales manager. The contract signing process is composed of the following steps: (1) The declaration from the sales manager owning the delegate authorization; (2) The client checking the information related to the declaration; (3) The challenge from the client; (4) The response from the sales manager; (5) The client verifying the response; (6) The sales manager providing the financial investment smart contract address; (7) The client checking the financial investment contract content; (8) The two parties signing the financial investment contract. If the client has a dispute on the financial investment contract, he/she can apply to the arbitration institution for dispute arbitration.

### Example scenario

Suppose the contract-issuing organization needs to manage its sales manager as the "signatory" delegate of the financial investment contract. First, it will create the financial investment smart contract and delegate authorization smart contract of the DBCS-SC and then publish them on the Ethereum blockchain. Using the delegate authorization smart contract, it can perform the function of adding a sales manager to the system as the financial investment contract signing delegate. The delegate authorization smart contract will include the sales manager’s Ethereum address and relevant delegate authorization information authorized to the sales manager, such as the delegate’s validity period and personalization. Then, before the client needs to check, pay and sign the financial investment contract, he/she will verify the unknow sales manager’s delegate authorization through the challenge-response agreement, for example, the unknow sales manager claims to have the delegate authorization of the contract-issuing organization. After the client verifies the identity of the unknown sales manager, the two parties can sign the financial investment contract. If the client has any objection to the signed financial investment contract, he/she can apply to the arbitration institution for dispute arbitration.

### Initialization

Assume that the contract-issuing organization, the sales manager and the client are all Ethereum users in the DBCS-SC. Ethereum users know how to create and publish smart contracts, perform smart contract functions, and execute transactions.

To initialize the DBCS-SC, the contract-issuing organization (*o*) creates a key pair of the public key (*EOA*) and the corresponding private key (*EPK*). The key pair will be used to build smart contracts and perform smart contract functions. Multiple options can be used to complete the key pair generation, including Ethereum wallet and online/offline Ethereum address generator. This paper uses *o*.*EPK* and *o*.*EOA* as the private key and public key of *o* respectively.

Then, *o* builds the smart contracts that will handle all functions. After building the smart contracts, *o* deploys them on the Ethereum blockchain under the corresponding smart contract addresses (*f*.*EOA* and *d*.*EOA*). The detailed information about the smart contracts can be accessed by checking the *f*.*EOA* and *d*.*EOA* in the blockchain, and their interfaces (*f*.*Interface* and *d*.*Interface*) can be created from the JSON Interface.

Then, *o* chooses the public medium (e.g.,website or public database) on which releasing *o*.*EOA*, *f*.*EOA*, *d*.*EOA*, *f*.*Interface* and *d*.*Interface* to make the public accessibility to them. The issuance of these information will act as evidence that *o* owns and manages *o*.*EOA*, *f*.*EOA* and *d*.*EOA*.

Equally, the sales manager (*s*) respectively creates a key pair of the private key and the public key as *s*.*EPK* and *s*.*EOA*. Alternatively, *o* can create the key pair (*i*.*e*., *s*.*EPK*, *s*.*EOA*) and send them to *s* through the safe communication channel.

### DBCS-SC’s smart contracts

The code in the financial investment smart contract mainly offers the following functions:

1. ContractPublishDeposit(): It can only be executed by a contract issuer who issue a financial investment contract by paying a deposit through this function.
2. AddContract(*o*.*EOA*, *o*.*contractName*, *o*.*contractContent*): It can only be executed by a contract issuer, so that the contract content can be added to the financial investment smart contract and the related information can be released. For example, a product can be recommended or several products can be recommended. The constraint can be the price of a product in a certain period of time and the correlation of several products in a combination. It takes the organization’s public key (*o*.*EOA*), contract name (*o*.*contractName*) and contract content (*o*.*contractContent*) as inputs. When this function is performed, the input and time stamp are output and the output is written to the financial investment smart contract.
3. ContractConfirmed(): It can only be executed by the client. It accepts an integer value as an input parameter. For example, 1 means that the client is satisfied with the financial investment contract, and 2 means that the client reports the financial investment contract fraud.
4. DisputeArbitration(*c*.*EOA*, bool): It can only be executed by an arbitration institution. The arbiter takes the Ethereum address of the client and the Boolean value of the ruling result as input. It is used to resolve fraud disputes in financial investment contracts.
5. SignedByEntity(*s*.*EOA*, *c*.*EOA*, *f*.*EOA*, *s*.*amount/c*.*amount*): It can only be executed by a sales manager/a client. The sales manager/the client can provide a certain commitment through the financial investment smart contract. For example, the client and the sales manager will invest a certain amount at a certain proportion, such as 9:1. If any clause is triggered, the amount in the contract (virtual currency) will be automatically transferred to the client/the sales manager without any third party intervention. It takes the sales manager’s public key (*s*.*EOA*), the client’s public key (*c*.*EOA*), the contract’s address (*f*. *EOA*) and the amount invested by the sales manager (*s*.*amount*)/the client (*c*.*amount*) as input. After executing this function, the input and timestamp will be output, and the output will be written to the financial investment smart contract.
6. ChangeStatus(): It can only be performed by the contract issuer to deactivate the financial investment smart contract.

The code in the delegate authorization smart contract mainly offers the following functions:

1. RequestDelegate(): It can only be executed by a sales manager who can send a delegate authorization request by paying a deposit through this function.
2. AddSalesManager(*s*.*EOA*, *s*.*delegate*, *s*.*note*): It can only be executed by the contract issuer, so that sales managers can be added to the delegate authorization smart contract and the corresponding delegates and related information can be released. It uses the public key of the sales manager (*s*.*EOA*), the delegate qualification authorized to *s* (*s*.*delegate*) and note (*s*.*note*) as input. The note can contain any other relevant information, such as validity date and personalization. When this function is executed, the input and timestamp are output, and the output is written to the delegate authorization smart contract.
3. RemoveSalesManager(*s*.*EOA*): It can only be executed by the contract issuer to remove sales managers from the delegate authorization smart contract and revoke their delegate authorization. It takes the public key of the sales manager (*s*.*EOA*) as input and deletes the sales manager from the delegate authorization smart contract after successful execution.
4. SalesManagerRefund(): It can only be executed by a sales manager. The sales manager can refund the deposit through this function. This means that the sales manager loses delegate authorization.
5. RequestSigning(): It can only be executed by a client requesting the signing of the financial investment contract.
6. ChangeStatus(): It can only be executed by the contract issuer to deactivate the delegate authorization smart contract.

### DBCS-SC’s contract signing process

When the sales manager requests to sign the financial investment contract with the client, the contract signing process will be executed. The process consists of the following steps:

1. Declaration: The sales manager *s* claims to the client *c* that he/she is the contract signing delegate of the contract-issuing organization *o*.
2. Information Check: To determine the details/information related to *s*.*EOA* and *o*.*EOA*, *c* checks the public medium where *o* released the *o*.*EOA*, *d*.*EOA*, and *d*.*Interface* it owns. Using these data, *c* is going to be able to access the *d* and check the information related to *s*.*EOA*, containing *s*.*delegate* and *s*.*note*. Given the data on the *d*, *c* is assured that the delegate qualification *s*.*delegate* is authorized by *o* to the owner of *s*.*EOA*. Currently, *c* can challenge *s* to verify whether he/she is the true owner of *s*.*EOA*.
3. Challenge: An arbitrary data *m* is chosen by *c* and *s* is requested to sign it.
4. Response: *s* signs *m* together with *s*.*EOA* and the private key *s*.*EPK*. The signature is defined by *Sig* = *Sign* (*s*.*EPK*, *s*.*EOA*, *m*), and thus a right *Sig* can only be established if *s* has *s*.*EPK*. *s* then sends *Sig* back to *c*.
5. Response Verification: After receiving the signature *Sig* from *s*, *c* will use the function *ResponseVerify* (*s*.*EOA*, *m*, *Sig*) for verification [38].
6. Contract Check: After the response verification is successful, *c* will verify the content of the financial investment smart contract and confirm whether *s* can sign the financial investment contract by accessing the delegate authorization smart contract.
7. Dispute Arbitration: After the contract content and the delegate authorization are verified, if there is no dispute in the financial investment contract, the two parties will sign the contract; if there is a dispute in the financial investment contract, the two parties will enter the dispute arbitration procedure.
8. Contract Signing: If there is no problem with the financial investment contract, both parties will complete the signing of the contract.

**Fig 3:**
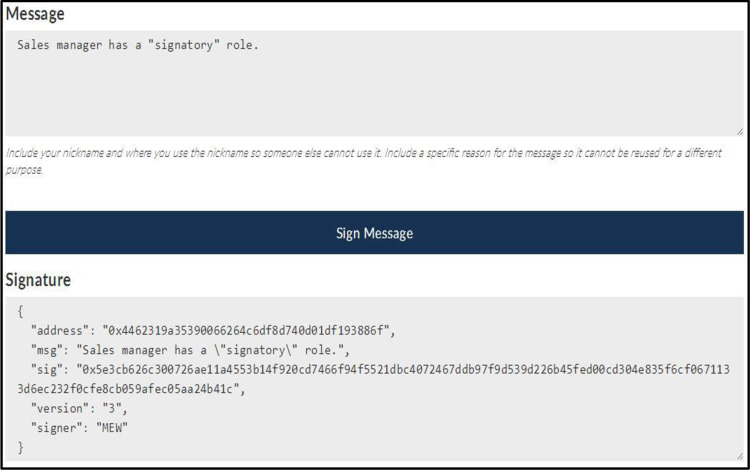
Signing a message.

**Fig 4:**
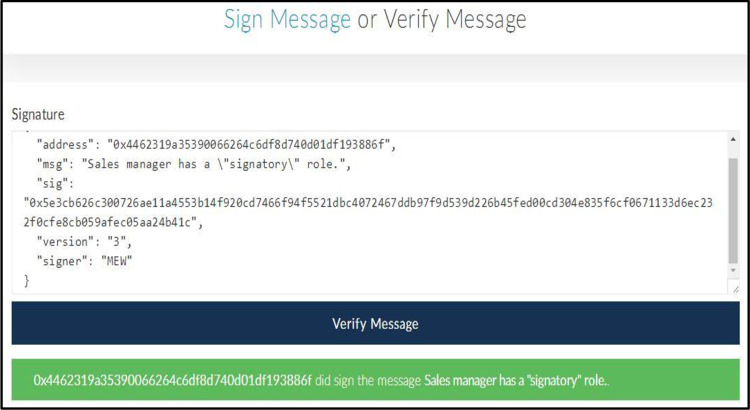
Verifying a signature.

The *eth*.*sign* (*address*, *web3*.*sha3(message)*) function that is the Ethereum source code can be used to create a signature [39]. The advantage of this function is that users can create signatures for messages without revealing the private key. Fig 3 shows an example of signing a message. Fig 4 shows an example of verifying a signature. These two examples are used to prove the ownership of *EOA*.

## Implementation of the smart contracts

### Sequence diagrams

The contract issuer pays the publishing contract deposit through the ContractPublishDeposit() function. If the sales manager agrees to the terms and conditions of the delegate authorization smart contract, he/she can request the delegate contract signing authorization by paying a deposit through the function RequestDelegate(). After the sales manager pays the deposit, the contract issuer executes the function AddSalesManager() to add the sales manager’s Ethereum address and the information related to the delegate contract signing to the list of sales managers in the delegate authorization smart contract, and broadcast the delegate authorization information to the blockchain network.

**Fig 5:**
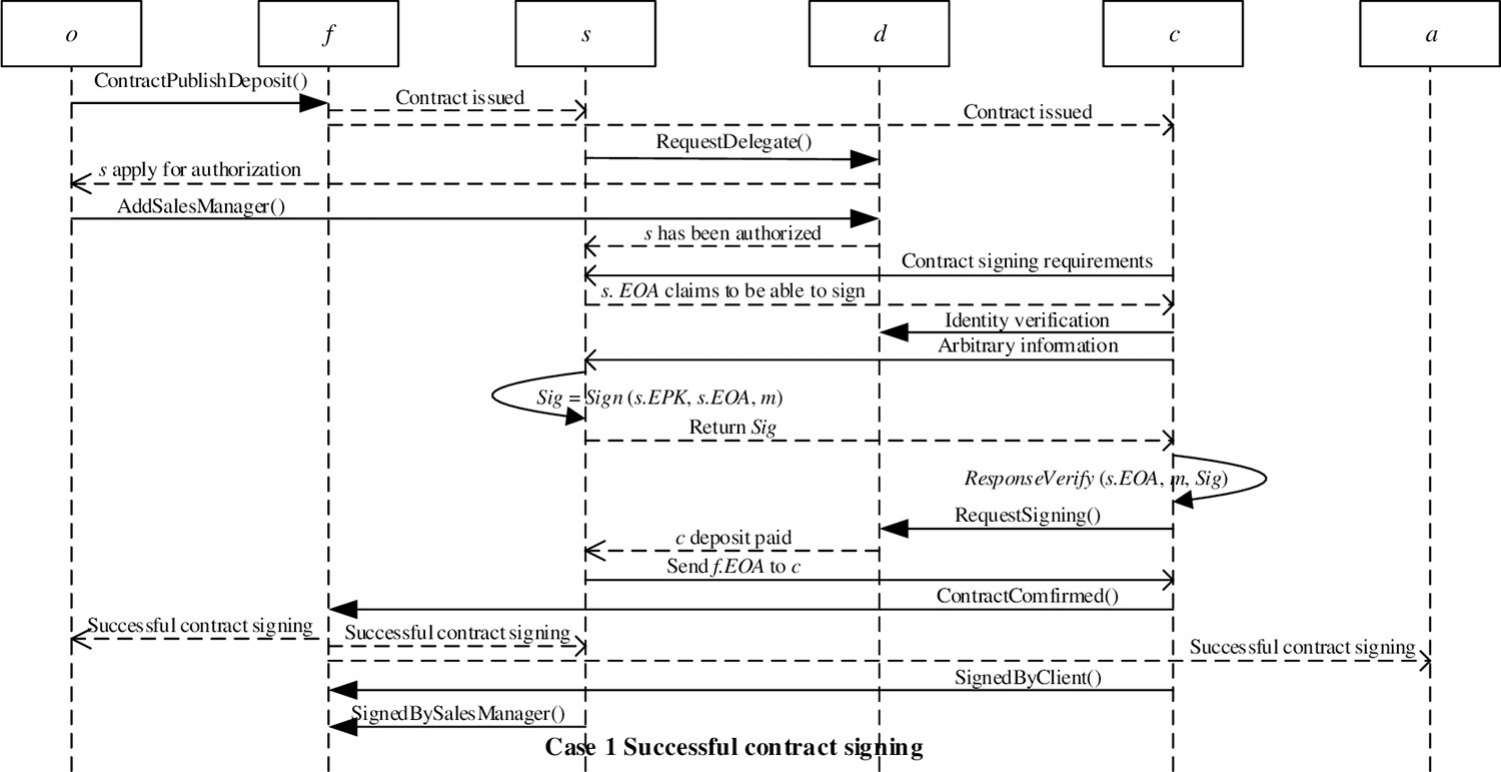
Successful contract signing.

The client broadcasts the information that needs to sign a financial investment contract to the blockchain network. The sales manager can respond to the client through his/her Ethereum address. The client can verify the authenticity of the response through the delegate authorization smart contract, and verify the digital signature of the sales manager through a challenge-response mechanism to confirm that the sales manager is the real owner of the Ethereum address. After verifying the identity of the sales manager, the client can request to sign the financial investment contract by paying a deposit through the RequestSigning() function in the delegate authorization smart contract. After the client pays the deposit, he/she will receive the financial investment smart contract Ethereum address *f*.*EOA* sent by the sales manager. If the client has no objection to the financial investment contract, the two parties will complete the contract signing. Fig 5 shows the successful signing of the financial investment contract.

If the client has objections to the financial investment contract, he/she can report contract fraud through the ContractConfirm() function. After the client confirms the report, the report information will be broadcast to the blockchain network. After the arbitration institution receives the report information, it will intervene in the dispute arbitration. The arbitration institution will determine whether there is fraud in the contract by checking the contract and referring to previous cases, and will give the ruling result by calling the DisputeArbitration() function.

**Fig 6:**
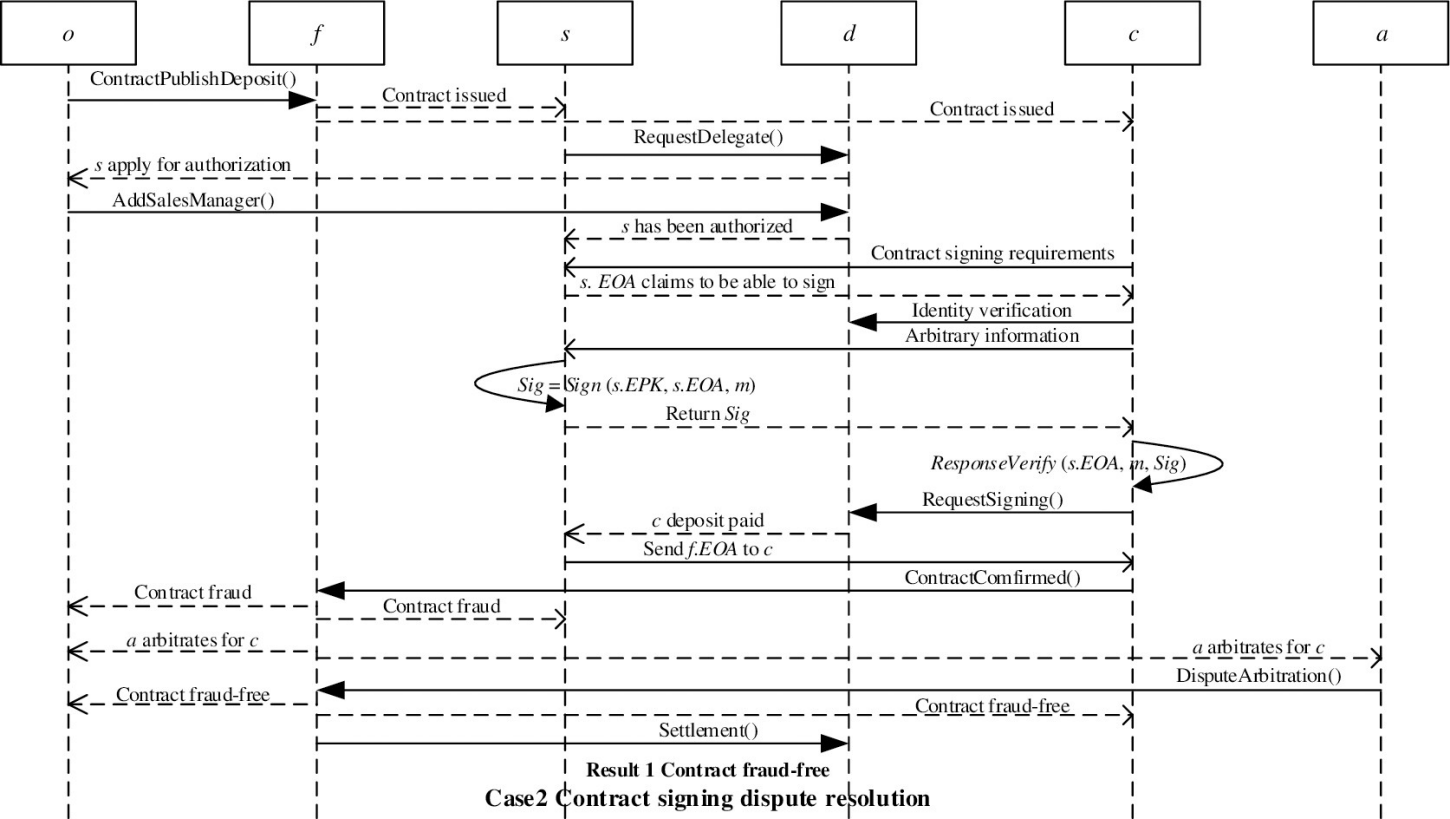
Contract fraud-free dispute resolution.

If the ruling result is that there is no fraud in the contract, the financial investment smart contract will automatically call the Settlement() function to punish the client’s false positives. Fig 6 shows the contract signing dispute resolution (the ruling result is that the contract is free of fraud).

**Fig 7:**
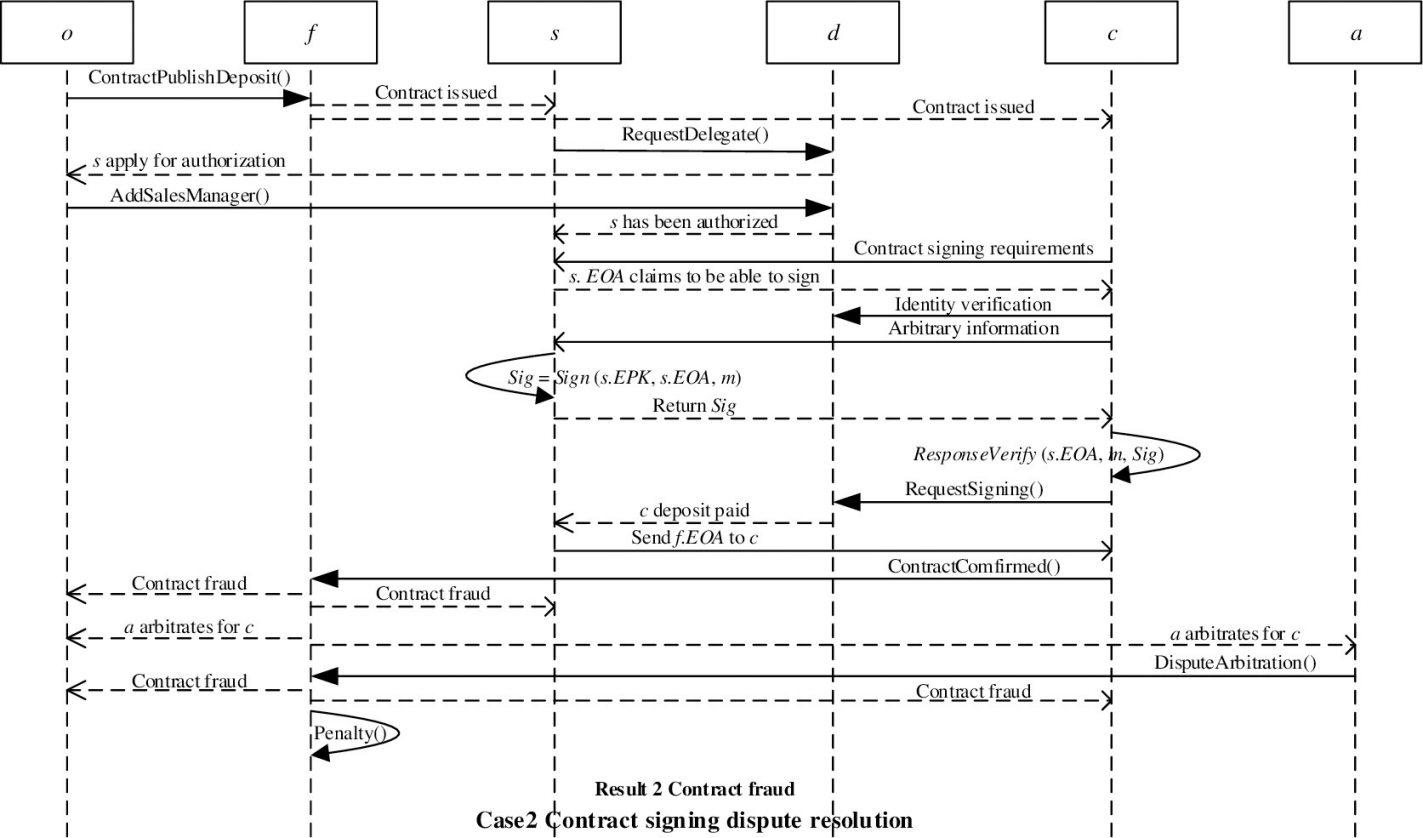
Contract fraud dispute resolution.

If the ruling result is contract fraud, the financial investment smart contract will automatically call the Penalty() function to punish the contract issuer’s fraudulent behavior. The ruling result will be broadcast to the blockchain network to avoid potential clients being defrauded by the contract. Fig 7 shows the contract signing dispute resolution (the ruling result is contract fraud).

### Algorithmic design

(1) Requesting contract signing algorithm

The client needs to request the financial investment contract signing service by paying a deposit to the delegate authorization smart contract. This is to ensure that the client’s behavior is honest and sincere. After the sales manager service is over, if the client is willing to sign a financial investment contract, the sales manager will send the Ethereum address of the contract to the client so that the client can access and verify the contract. Algorithm 1 shows the algorithm for a client to request a contract signing service.

**Algorithm 1 Request contract signing**

**Input:** *EOA*, cDeposit, dStatus, cStatus

**Output:** *f*.*EOA*

**1:** *EOA* ← Ethereum address.

**2:** cDeposit ← deposit value.

**3:** dStatus ← delegate authorization smart contract status.

**4:** cStatus ← client status.

**5: if** msg.value = cDeposit **then**

**6:    if** dStatus = waiting for *c* **then**

**7:** *c*.EOA.send(msg.value). //*c* has paid the deposit

**8:        ** cStatus ← cPaid. //cStatus status is cPaid

**9:        ** Create a notification that *c* has paid the deposit.

**10:        ** Create a notification that the *f*.*EOA* address has been sent to *c*.

**11:    else**

**12:        ** Revert the delegate authorization smart contract status and display errors.

**13:    end if**

**14: end if**

(2) Successful contract signing algorithm

After the client accesses and verifies the financial investment smart contract through the Ethereum address of the contract, if the client confirms that the contract is a satisfactory contract, he/she can complete the contract signing through the financial investment smart contract, then the contract signing is successful. Algorithm 2 shows the algorithm for successful financial investment contract signing.

**Algorithm 2 Successful contract signing**

**Input:** *EOA*, cStatus, cPaid, aReceivedReport

**Output:** Contract signed successfully

**1:** *EOA* ← Ethereum address.

**2:** cStatus ← *c* status.

**3:** cPaid ← cStatus value.

**4:** *a*ReceivedReport ← cStatus value.

**5: if** cStatus = = cPaid or cStatus = = aReceivedReport **then**

**6:    if** cStatus = = cPaid **then**

**7:**        Create a notification that the contract is signed successfully.

**8:    else**

**9:        if** cStatus = = aReceivedReport **then**

**10:            ** Execute DisputeArbitration().

**11:            ** Turn to Algorithm 3.

**12:        else**

**13:**            Revert the financial investment smart contract status and display errors.

**14:        end if**

**15:    end if**

**16: else**

**17:**    Revert the financial investment smart contract status and display errors.

**18: end if**

(3) Contract fraud dispute resolution algorithm

If the client confirms that the contract is contract fraud, the arbitration institution will intervene in the arbitration through the financial investment smart contract. The arbitration institution will decide whether there is fraud in the contract by checking the contract and referring to previous similar cases. If the ruling result is contract fraud-free, the client will be penalized. If the ruling result is contract fraud, the contract issuer will be fined.

**Algorithm 3 Contract fraud dispute resolution**

**Input:** *EOA*, *Res*, cStatus, DisputeRuling

**Output:** Ruling result.

**1:** *EOA* ← Ethereum address.

**2:** *Res* ← ruling result.

**3:** cStatus ← *c* status.

**4:** DisputeRuling ← cStatus value.

**5: if** cStatus = = DisputeRuling **then**

**6:    if** *Res* = = false **then**

**7:**        *Res* ← contract fraud-free.

**8:**        Execute Settlement().

**9:    else**

**10:**        *Res* ← contract fraud.

**11:**        Execute Penalty().

**12:    end if**

**13: else**

**14:**        Revert the financial investment smart contract status and display errors.

**15: end if**

## Testing and validation of the smart contracts

The writing and compilation of smart contracts will use Solidity and Remix IDE, and the simulation of smart contracts will be based on Ethereum blockchain and wallet. The simulation of financial investment smart contract and delegate authorization smart contract is achieved by simulating the main functions of smart contract. The validity of the model and algorithm is proved through the generated event occurrence graph combined with the smart contract issuance and signing process. The main function of the delegate authorization smart contract is that the client requests financial investment contract signing service; the main function of the financial investment smart contract is successful contract signing and dispute resolution of contract fraud.

### Test preparation

In the Ethereum blockchain, the Ethereum addresses of all participants are as follows:

The contract issuer’s Ethereum address:

“0xD14EccFd8251f9dEAa78c1c09f399235E4bc472e”

The sales manager’s Ethereum address:

“0x236DA1E0726522B76d5F3C1A3B4Ed40E59afD9DD”

The client’s Ethereum address:

“0xbDc1eA1b68Cff0718F159eA1268CB41188a9fa60”

The arbiter’s Ethereum address:

“0xc9Adba823dF6A34b9F2C7591b0699a4aF7611BC1”

The functions of a smart contract are performed by a specific entity, which is authorized by the Solidity modifier and the Ethereum address uniqueness. If an entity without a specific Ethereum address requests to execute a function of the smart contract, the function execution will fail and the calling behavior will not be recorded in the smart contract and the blockchain.

### Validation of the main functions

1. Request contract signing

**Fig 8:**
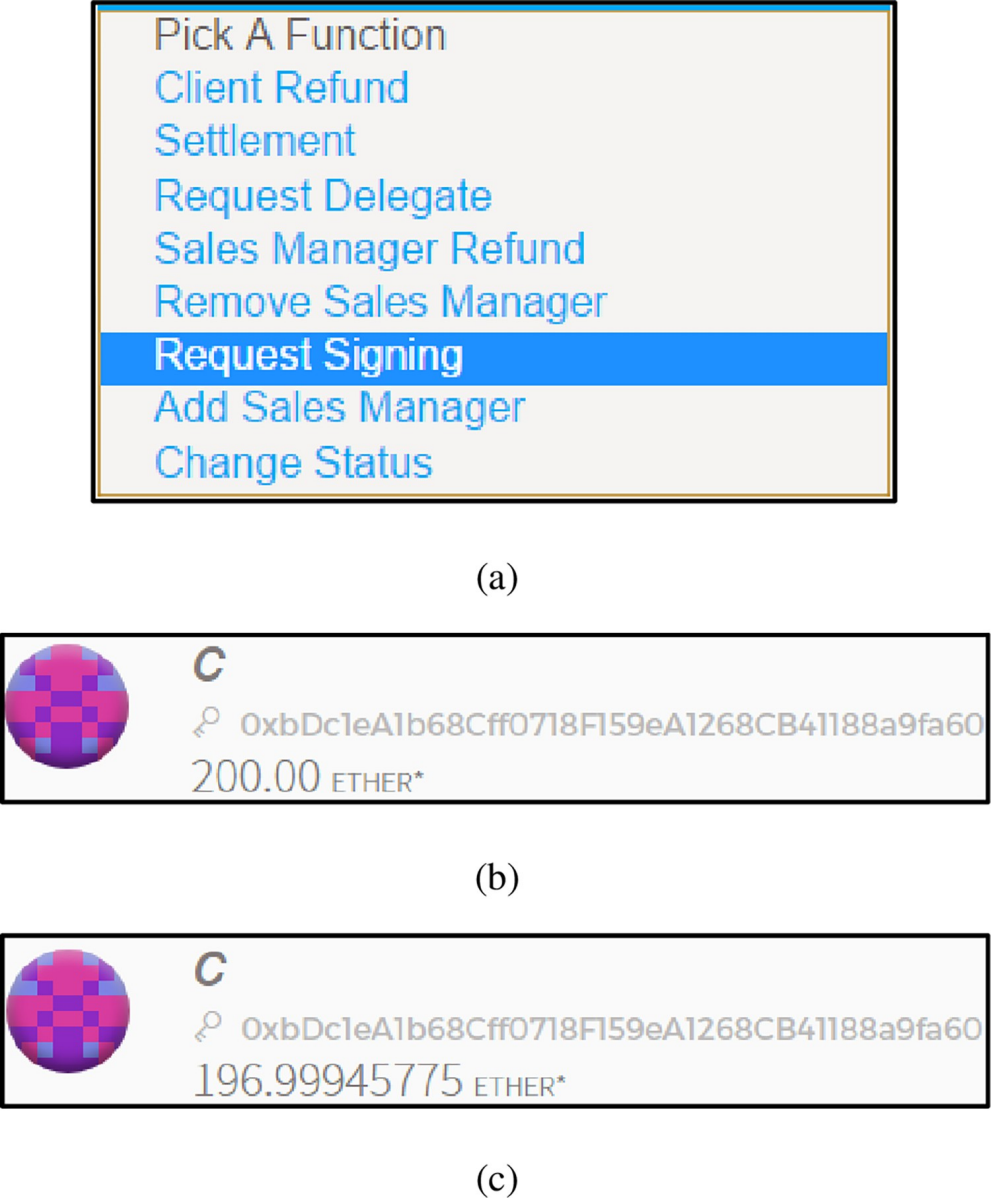
(a). A list of delegate authorization smart contract functions. (b). The original account balance of the client. (c). The current account balance of the client.

The client uses the RequestSigning() function to send a financial investment contract signing service request to the delegate authorization smart contract. Fig 8(A) shows a list of delegate authorization smart contract functions. Fig 8(B) shows the original account balance of the client. Fig 8(C) shows the current account balance of the client. Since the smart contract function called needs to be paid for gas, the client’s account balance is less than 197 Ether, which indicates that the client has successfully sent a financial investment contract signing service request.

From the results shown in Fig 8, it can be seen that the "delegate authorization smart contract" and "requesting contract signing algorithm" in the delegate contract signing mechanism are feasible and effective.

1. Successful contract signing

**Fig 9:**
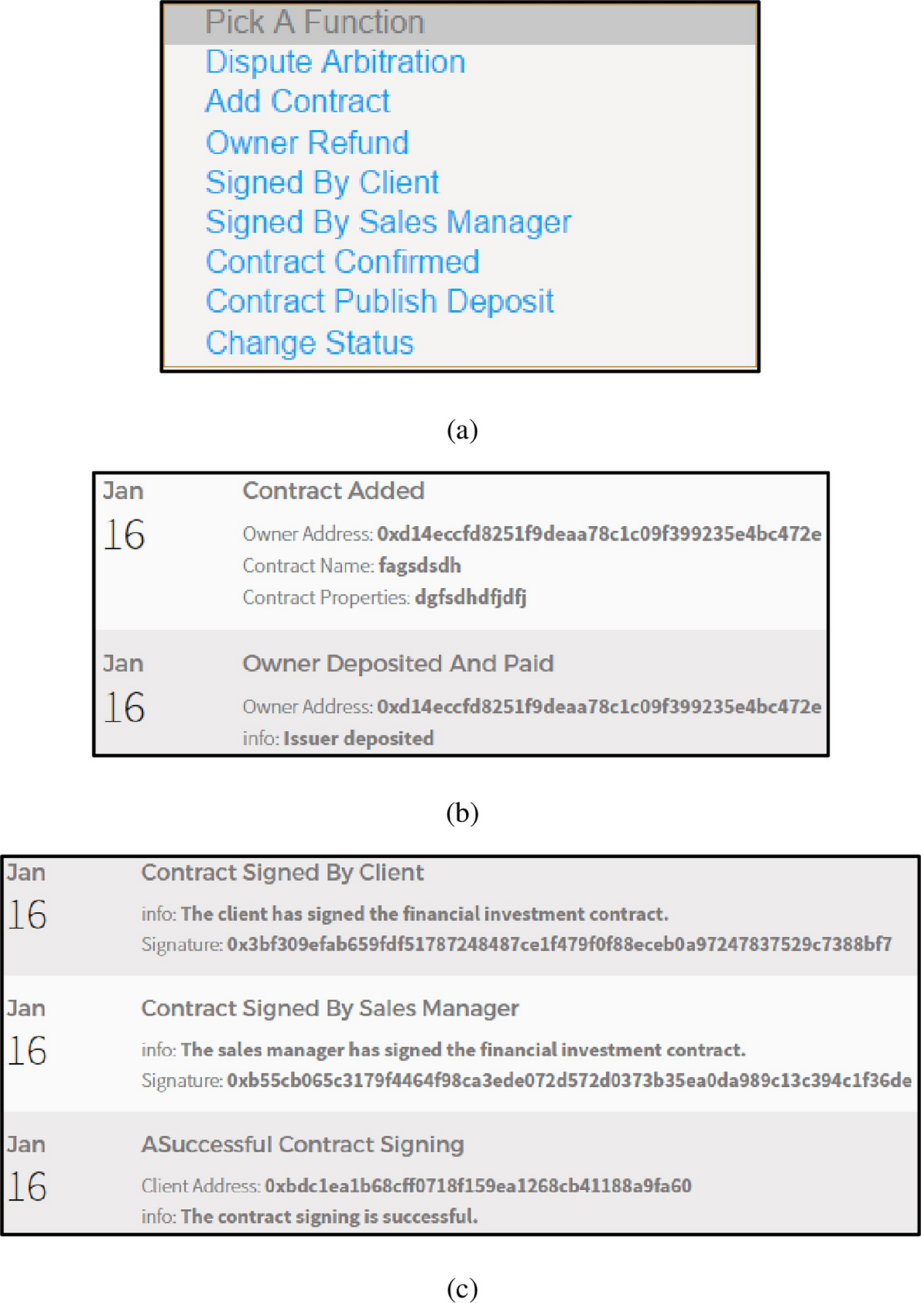
(a). A list of financial investment smart contract functions. (b). The successful release of the financial investment contract. (c). The success of contract signing.

The client uses the ContractConfirmed() function to confirm whether the contract signing is successful. Fig 9(A) shows a list of financial investment smart contract functions. Fig 9 (B) shows the successful release of the financial investment contract. Fig 9(C) shows the success of contract signing.

From the results shown in Fig 9, it can be seen that the "financial investment smart contract" and "successful contract signing algorithm" in the delegate contract signing mechanism are feasible and effective.

1. Contract fraud dispute resolution

**Fig 10:**
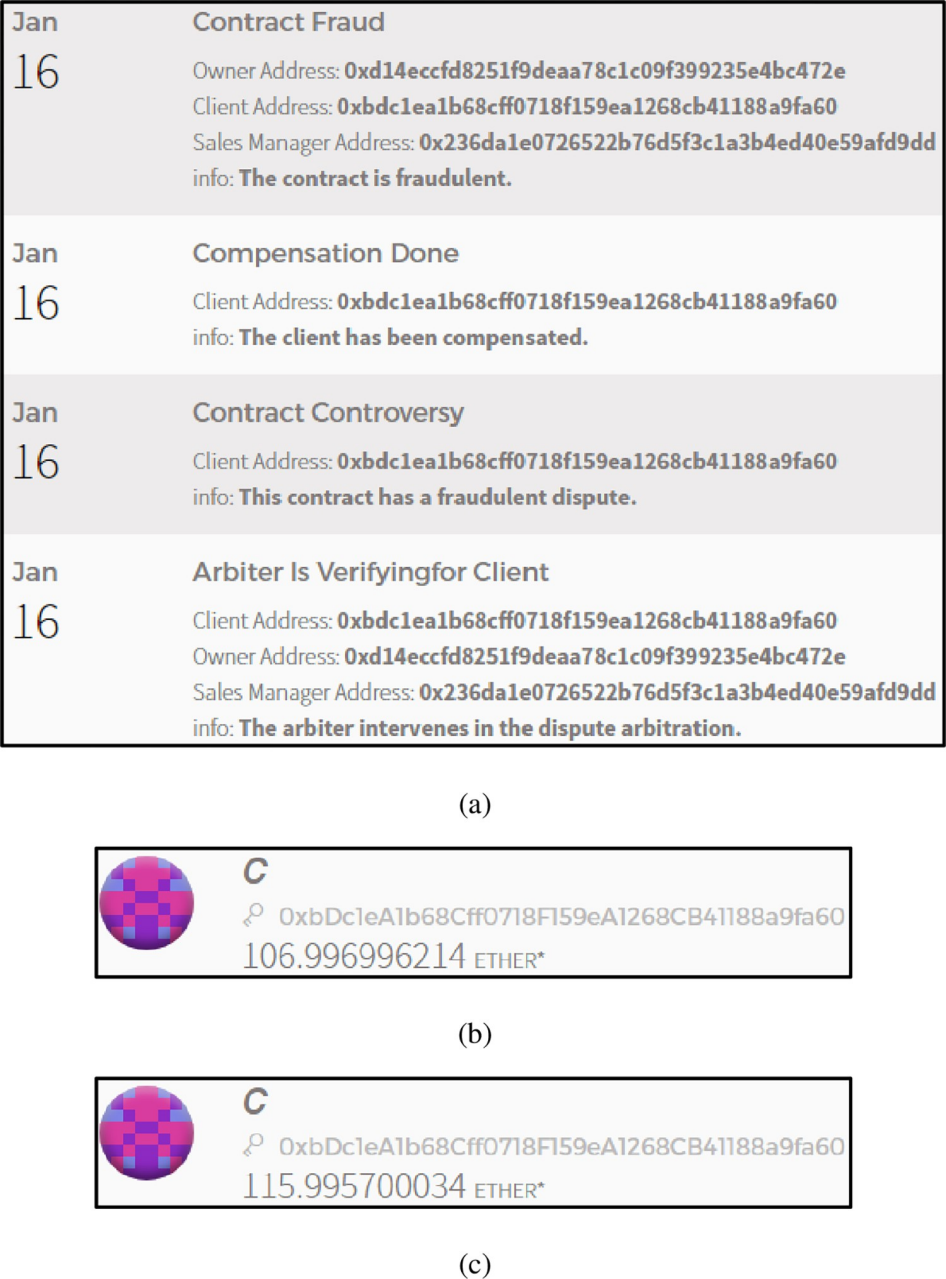
(a). The occurrence of a contract fraud compensation event. (b). The original account balance of the client. (c). The current account balance of the client.

If the client uses the ContractConfirmed() function to confirm that the contract is fraudulent, the arbiter will intervene in the dispute. If the ruling result is contract fraud, the financial investment smart contract will automatically complete the settlement of the compensation client. On the other hand, if the ruling result is that the contract is free of fraud, the client will also be fined for malicious reporting. Fig 10(A) shows the occurrence of a contract fraud compensation event. Fig 10(B) shows the original account balance of the client. Fig 10(C) shows the current account balance of the client.

From the results shown in Fig 10, it can be seen that the "contract fraud dispute resolution algorithm" in the delegate contract signing mechanism are feasible and effective.

## Discussions and limitations

### Reasons for choosing smart contracts

Since the contract issuer is the direct beneficiary of the financial investment contract, there is a problem of contract fraud in order to seek greater benefits. This is the reason for the construction of the delegate contract signing mechanism based on smart contracts (financial investment smart contract and delegate authorization smart contract).

Smart contracts have the following advantages:

1. Smart contracts have the functions of receiving, processing, storing and sending information, and can also control assets and respond to externally received information, which is essentially an automated computer program.
2. Smart contracts are a program running on the blockchain, and the blockchain has key features such as decentralization, tamper-proof, traceability, and anonymity, which make smart contracts also have these features.
3. The characteristics of smart contracts can enable participating nodes on the blockchain to receive timely information on whether there is fraud in the contract, so that participating nodes can quickly feedback their opinions on contract fraud.

A (smart contract)-based delegate contract signing solution is proposed to solve the problem of contract fraud caused by information and interest asymmetry. This solution is divided into three stages. In the first stage, "delegate authorization smart contract" and "requesting contract signing algorithm" are designed to achieve the goal of "contract signing request". In the second stage, "financial investment smart contract" and "successful contract signing algorithm" are designed to achieve the goal of "contract signing". In the third stage, "contract fraud dispute resolution algorithm" is designed to achieve the goal of "dispute resolution". Three stages are completed, and the contract fraud problem is solved. This provides a new method for solving the problem of contract fraud caused by information and interest asymmetry, as well as a new choice for contract fraud prevention and supervision.

### Performance analysis

The framework and different characteristics of DBCS-SC are analyzed in this section. It provides a safe and effective DBCS mechanism, which can use smart contracts to sign contracts. In addition, the use of blockchain also provides other characteristics that are different from traditional contract signing characteristics.

(1) Issuance

In the DBCS-SC, the use of smart contracts can safely and effectively execute the issuance of the financial investment contract, the assignment of delegate authorization and other related information.

① Contract Content

The financial investment contract is an official text with legal effect, so the content is standardized and rigorous, for example, the contract between the two parties is signed. The main contents of the contract include the entrusted matters, investment requirements, duration, Party A’s responsibilities and obligations, Party B’s responsibilities and obligations, fees and payment methods, breach of contract liability, confidentiality, mutual commitment, dispute settlement, contract validity, etc.

② Personalization

The relationship between the sales manager and the delegate authorization is represented by the ownership of the private key, which corresponds to the authorized delegate’s public key. In this way, there is a possibility of security risks, namely leakage and loss of keys. The DBCS-SC has personalized features to prevent key leakage. The personalization can be directly attached to the sales manager through the "note" part of the AddSalesManager() function. For example, the contract-issuing organization can write a note "The signatory delegate is given to the sales manager with ID 3261". This personalization will allow the sales manager to be more cautious when revealing his/her private key, because a maliciously exploited principal can be subsequently investigated.

③ Delegate Reauthorization

If the private key, digital wallet and public key are forgotten or lost. Without the corresponding unique private key, it is impossible to prove the ownership of *EOA*. The DBCS-SC has a revocation feature to reduce the risk of key loss. The contract-issuing organization can easily delete the lost *EOA* through the RemoveSalesManager() function and reauthorize the delegate to the new *EOA* of the sales manager.

④ Term of Validity

The Ethereum timestamp server provides a natural solution that can include a validity period or a delegate authorization validity period in the proposed system. The contract-issuing organization may include the validity period of the delegate authorization to be issued in the "note" section of the AddSalesManager() function. Then, the client can check the validity of the declared delegate based on the timestamp authorized by the organization to the sales manager.

⑤ Accounts Processed

The contract-issuing organization only needs to create and process one account (*o*.*EOA*, *o*.*EPK*). It can be used to create smart contracts and perform their functions. Equally, the sales manager only needs to create and process one account (*s*.*EOA*, *s*.*EPK*). It can be used to receive delegate authorization and sign financial investment contract. The client only needs to create and process one account (*c*.*EOA*, *c*.*EPK*). It can be used to sign financial investment contract.

(2) Management

The contract-issuing organization can use the AddSalesManager() function to update the information related to the sales manager’s delegate authorization. Even if the information is easy to update, anyone can verify all updates, so it will be done in a responsible way.

(3) Revocation

The RemoveSalesManager() function is used to implement the delete function, which will delete the sales manager’s information from the DBCS-SC. If the delegation authorization no longer belongs to the sales manager, this function will work to prevent the sales manager from claiming to own the delegation authorization.

(4) Verification

The contract signing process of DBCS-SC includes the challenge-response agreement, which is designed to securely verify the sales manager’s delegate authorization. The client can verify the financial investment contract content and the sales manager’s signature qualification by accessing the financial investment smart contract and the delegate authorization smart contract.

(5) Transparency

Due to all functions performed in the smart contract are reflected in the event log of the smart contract and the Ethereum blockchain, DBCS-SC achieves the property of transparency. Therefore, if the other entity does not know it, the entity will not be able to perform "secret" actions. Furthermore, the entity will not bear any responsibility (because only the relevant entity can control its private key).

(6) Anonymity

Blockchain participating nodes can only query the transfer records of all nodes on the blockchain network, but they have no ability to know who the specific entity behind the transfer address is. Therefore, the transparency of investment amount, total assets, contract signing and other information will not cause the risk of privacy leakage.

(7) Restriction

Due to the smart contract includes modifiers in its source code, the DBCS-SC implements the restriction function. Thus, the specific functions of different entities will be restricted. For instance, a smart contract has an "onlyOwner" modifier. It only allows the creator/owner of the contract to execute the AddContract(), AddSalesManager(), RemoveSalesManager(), and ChangeStatus() functions. If these functions are executed by a non-owner, the execution will fail and the operation behavior will not be recorded on the DBCS-SC and blockchain.

(8) The differences from traditional contract signing

Table 1 shows the difference between (smart contract)-based delegate contract signing and traditional delegate contract signing.

**Table 1:** The comparison between (smart contract)-based delegate contract signing and traditional delegate contract signing.

Characteristic	Traditional contract signing	(Smart contract)-based contract singing
**Signature form**	Electronic/Handwritten	Digital
**Signing mode**	Centralized	Decentralized
**Data robustness**	Tamperable	Non-tamperable
**Media-based**	Internet/Paper	Blockchain
**Transparencity**	Intransparent	Transparent
**Payment manner**	Third-party	Smart contract
**Traceability**	Untraceable	Traceable
**Auditability**	Unauditable	Auditable
**Accountability**	Unaccountable	Accountable
**Anonymity**	Non-anonymous	Anonymous

### Strengths and weaknesses

A delegate contract signing mechanism based on smart contracts is proposed. In this mechanism, the financial investment contract is designed as a financial investment smart contract, and the delegate authorization contract is designed as a delegate authorization smart contract. Considering the non-tamperability, transparency and traceability of data, the traditional delegate contract signing mechanism is powerless to eliminate the risk of contract fraud. In contrast, considering the delegate in contract signing, the delegate contract signing mechanism proposed in this paper ensures the anti-contract fraud of contract signing. Furthermore, the blockchain has the characteristics of decentralization, traceability, and tamper resistance, which helps to establish a contract fraud prevention mechanism on the basis of eliminating information asymmetry, and at the same time eliminates the fraud risk caused by interest asymmetry. Moreover, the ruling results obtained by the proposed dispute resolution mechanism and algorithm are accurate results, which resolves contract fraud disputes simply and effectively.

Since this paper is the first research to propose a delegate contract signing mechanism based on smart contract, the maturity of the mechanism in practice needs to be further verified. Furthermore, the sharp fluctuation of cryptocurrency value and regulatory uncertainty will cause the sharp fluctuation of financial investment contract value, which increases the risk of income uncertainty. Stable currency is a possible solution to the volatility of cryptocurrency. It is a cryptocurrency linked to the value of subject assets such as legal currency or financial assets. This ensures that the value of the stable currency is consistent. Moreover, arbiters may be compromised to rule against the principles of fairness and justice in contract fraud disputes. One possible solution to this limitation is to establish a reasonable reward and punishment system to ensure that arbiters will resolve contract fraud disputes fairly and impartially.

## Conclusion and future scope

In this paper, a delegate contract signing solution is proposed to solve the contract fraud problem. Firstly, the delegate contract signing solution from "contract signing request", "contract signing" to "dispute resolution" is realized through the construction of a delegate contract signing mechanism. Next, the authorization of contract signing is controlled, penalties are set and disputes are resolved through the design of "requesting contract signing", "successful contract signing" and "contract fraud dispute resolution" algorithms. Furthermore, the code writing and debugging of smart contracts are done through the Remix IDE. Moreover, the testing and verification of smart contracts are achieved through the Ethereum blockchain and wallet. Finally, the performance, advantages and disadvantages of the solution are discussed.

The future work area is the application research of blockchain in the field of financial technology. For example, financial investment trust mechanism based on smart contract.
